# NRF2 and glutathione are key resistance mediators to temozolomide in glioma and melanoma cells

**DOI:** 10.18632/oncotarget.10129

**Published:** 2016-06-17

**Authors:** Clarissa Ribeiro Reily Rocha, Gustavo Satoru Kajitani, Annabel Quinet, Rodrigo Soares Fortunato, Carlos Frederico Martins Menck

**Affiliations:** ^1^ Department of Microbiology, Institute of Biomedical Sciences, University of São Paulo, São Paulo, Brazil; ^2^ Institute of Biophysics Carlos Chagas Filho, Federal University of Rio de Janeiro, Rio de Janeiro, Brazil

**Keywords:** temozolomide, resistance, glioma, melanoma, NRF2

## Abstract

Cancer is a leading cause of death worldwide, and while great advances have been made particularly in chemotherapy, many types of cancer still present a dismal prognosis. In the case of glioma, temozolomide (TMZ) is the main option for treatment, but it has limited success due to drug resistance. While this resistance is usually associated to DNA repair mechanisms, in this work we demonstrate that oxidative stress plays an important role. We showed that upon TMZ treatment there is an induction of the nuclear factor erythroid 2-related factor 2 (NRF2), which is the main antioxidant transcription factor regulator in human cells. This is accompanied by an enhancement of glutathione (GSH) concentration in the tumor cells. The effectiveness of this pathway was proven by silencing NFR2, which greatly enhanced cell death upon TMZ treatment both *in vitro* and *in vivo*. Also, higher DNA damage and induced cell death was observed by combining BSO - a GSH inhibitor - with TMZ. Similar effects were also observed using *in vitro* and *in vivo* models of melanoma, thus possibly indicating that GSH has a decisive role in TMZ resistance in a wider range of tumors. Thus, a combined regimen of BSO and TMZ configures an interesting therapeutic alternative for fighting both glioma and melanoma.

## INTRODUCTION

Malignant gliomas are the most common type of primary brain tumors in adults, with an incidence rate of approximately 5 cases per 100,000 inhabitants [1]. It is also one of most aggressive types of cancer. Patients diagnosed with glioma have a dismal prognosis, with a median survival rate of 15 months and a 5-year survival rate of ~2% [2]. Current therapy includes surgery for tumor resection, followed by radiotherapy and/or concomitant adjuvant chemotherapy. The main chemotherapy protocol for this type of tumor is based on temozolomide (TMZ) [3].

Metastatic melanoma shares several of glioma's features, in particular, high aggressiveness and poor prognosis. The average survival rate for melanoma patients with brain metastasis is about 4 months and a complete cure is observed in less than 1% of the patients [4]. Besides surgery and radiotherapy, melanoma patients are usually submitted to chemotherapy treatment with dacarbazine (DTIC), fotemustine or cisplatin [5], and, as it is the case with glioma, TMZ.

Nevertheless, as revealed by glioma and melanoma patients' average survival rates, current chemotherapeutic protocols have limited success. This occurs mainly due to drug resistance. Several mechanisms command resistance and many of those are tissue and/or drug specific. Thus, it is crucial to fully understand chemotherapy resistance mechanisms in order to develop new approaches to overcome it, improving the efficacy of therapy protocols.

Temozolomide (TMZ) is an alkylating agent that causes methylation on DNA bases in several positions, ultimately leading to cell death. Many DNA repair mechanisms are involved in resolution of DNA damage induced by TMZ, such as base excision repair (BER), mismatch repair (MMR) and direct repair by O^6^- methylguanine-DNA methyltransferase (MGMT). In fact, until now, the main known TMZ resistance mechanisms are related to the DNA repair capacity of the cells [6]. However, its is important to notice that due to poor drug response or tumor relapse observed upon TMZ treatment it is reasonable to speculate that other mechanisms are involved in drug resistance. In this context, it was recently shown that clinical achievable doses of TMZ induced high levels of mitochondrial DNA damage in human myeloid precursor cells [7]. Furthermore, Zhang et al. (2010) demonstrated for the first time that the treatment of glioblastoma cells with TMZ increased reactive oxygen species (ROS) levels. This was related to the activation of AMP-activated protein kinase, leading to cellular apoptosis [8]. Thus, oxidative stress induced upon TMZ treatment may play an important role in cell death induced by this drug.

Nuclear factor erythroid 2-related 2 (NRF2) is well known as the master regulator of antioxidant response, maintaining redox homeostasis in the cells [9]. Under physiological conditions NRF2 binds to KEAP1 (Kelch-like ECH associated protein 1), which directs NRF2 continuously to proteasome degradation. However, in oxidative stress situations, KEAP1 is oxidized and NRF2 is readily translocated into the nucleus where it can activate many different genes [10]. Among those, NRF2 controls the expression of two enzymes responsible for glutathione (GSH) synthesis, namely Glutamate-cysteine ligase modifier subunit (GCLM) and Glutamate-cysteine ligase catalytic subunit (GCLC), and also enzymes related to GSH utilization such as glutathione redutase, glutathione peroxidase and glutathione S-transferase (GST) [11].

GSH is a highly abundant, low-molecular-weight peptide in the cell, that plays a critical role in maintaining the cellular redox balance, acting as a free radical scavenger [12]. Additionally, GSH has a protective role against xenobiotic agents due to its highly reactive thiol group binds that inactivates those agents [13]. In fact, the GSH content and GST activity have long been associated with chemotherapy resistance in numerous cell lines and tumor tissues [14,15,16].

In this work, using either TMZ-sensitive or resistant glioma cell lines, we observed that NRF2 plays a crucial role in TMZ resistance. We showed that the transcriptional factor NRF2 mediates TMZ resistance through GSH synthesis and utilization. NRF2 silencing greatly sensitized glioma cells to TMZ both *in vitro* and *in vivo*. Importantly, GSH depletion by L-buthionine [S,R]-sulfoximine (BSO), a GSH-synthesis inhibitor, strongly potentiated TMZ-induced DNA damage and cell death in glioma and melanoma cell lines (*in vitro* and *in vivo*). Thus, the combination of BSO with TMZ is proposed as an extremely powerful approach to improve chemotherapy efficacy in both tumors, providing a new exciting alternative to treat these types of neoplasia.

## RESULTS

### TMZ induces NRF2 expression

**Figure 1:**
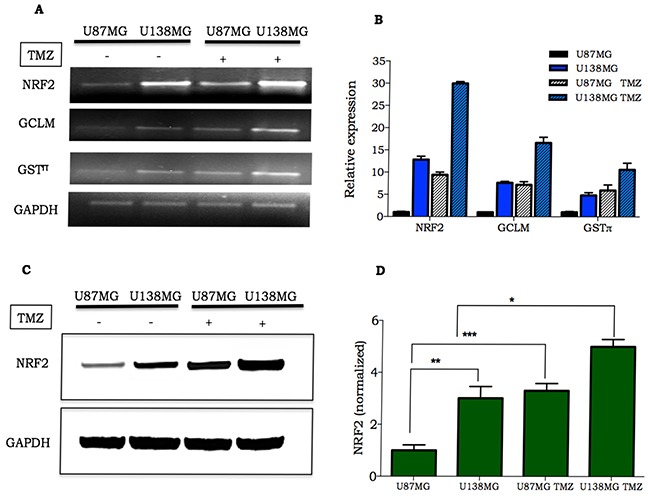
Expression of NRF2 and its target genes in glioma cell lines **A-B.** Representative image and quantification of NFR2, GCLM and GSTπ mRNA in U138MG and U87MG at basal level or 4 h after TMZ treatment (300 μM); **C.** NRF2 detection in glioma cells untreated or treated with 300 μM TMZ. Samples were collected 24 h after TMZ treatment and analyzed by western blot; **D.** Quantification of NRF2 protein expression in U87MG and U138MG submitted or not to TMZ treatment. Data were normalized by GAPDH expression followed by normalization by NRF2/GAPDH ratio verified on untreated U87MG cell line. Values are mean ± *SEM* of three independent experiments, *P< 0.05, **P< 0.01, ***P< 0.001.

NRF2 plays a crucial role on protective response against oxidative agents especially through induction of GSH synthesis [9]. In a previous work, we observed a significant difference in GSH levels between glioma cell lines [17], leading us to hypothesize that this could be due to *NRF2* differential gene expression. In fact, real time PCR analysis indicated that the U138MG, when compared to the U87MG cell line, displayed higher *NRF2* mRNA expression. Similarly, higher levels of mRNA expression were observed for NRF2 target genes, such as the glutamate cysteine ligase modifier subunit (*GCLM)* and glutathione S-transferase (*GSTπ)*, involved in GSH synthesis and utilization, respectively (Figure 1A-1B). Furthermore, TMZ treatment elicited a robust induction of *NRF2*, *GCLM* and *GSTπ* mRNA in the two glioma cell lines (Figure 1A-1B). Different levels of NRF2 between cells lines and TMZ-induction of NRF2 were confirmed for protein expression, by western blot analysis. As shown in Figure 1C-1D, NRF2 protein expression was 3-fold higher at basal levels in U138MG cells in comparison to U87MG cells. Moreover, NRF2 expression increased 3-fold in U87MG and 2-fold in U138MG cell lines upon TMZ treatment.

### NRF2 induces GSH synthesis as a protective mechanism upon TMZ treatment

**Figure 2:**
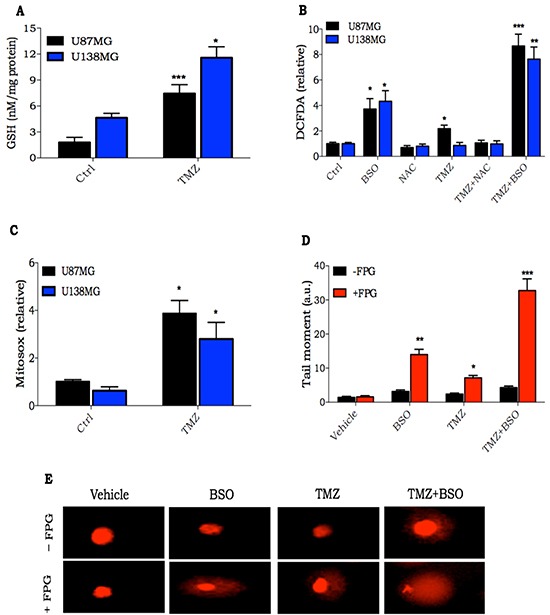
Consequences of oxidative stress induction after TMZ treatment **A.** Intracellular GSH quantification in glioma cells treated with TMZ (300 μM) for 24 h; **B.** Glioma cells were pre-incubated with BSO (100 μM) or NAC (1 mM) for 16 h and then treated with TMZ (300 μM) for 2 h. ROS was detected by DCFDA probe, and analyzed by flow cytometry; **C.** Quantification of mitochondrial O_2_^−^ generation using MitoSOX Redin glioma cells treated withTMZ (300 μM) for 2 h; **D-E.** Quantification and representative image of alkaline comet assay of U138MG glioma cells treated with TMZ alone (300 μM for 2 h) or in combination with BSO (100 μM, pre-incubated for 16 h). Quantification was done by measuring tail length of cells nuclei incubated or not with FPG endonuclease. Values are mean ± *SEM* of three independent experiments, *P< 0.05, **P< 0.01, ***P< 0.001.

Next, we measured the intracellular GSH levels in U87MG and U138MG cells submitted or not to TMZ treatment. As previously described, U138MG cell line has a higher GSH level when compared to U87MG. Moreover, TMZ treatment (24 h) was able to triple and double GSH levels in U87MG and U138MG, respectively (Figure 2A).

In order to evaluate the role of GSH in TMZ resistance, we modulated GSH levels using BSO or N-acetyl cysteine (NAC), a GSH synthesis inhibitor and precursor, respectively. As GSH is crucial to maintain redox homeostasis, we measured intracellular ROS levels in cells pre-treated with BSO or NAC, treated or not with TMZ for two hours. Although there was a significant increase in ROS levels when cells were treated with BSO, the levels were much higher when treatment was performed with TMZ in combination with BSO. Furthermore, NAC was able to inhibit the small TMZ ROS induction (Figure 2B). To examine possible sources of ROS induced after treatment with TMZ, acute mitochondrial ROS formation was measured using MitoSOX Red. Quantitative analysis indicated that TMZ treatment significantly increased mitochondrial production of ROS (Figure 2C).

Next, nuclear DNA damage from ROS generated after TMZ treatment for 2 h was evaluated. Thus, we performed a modified alkaline comet assay using the FPG enzyme. FPG is a DNA glycosylate that identifies oxidized guanines, such as 8-oxoguanine, on the DNA molecule. It cleaves at the N-glycosydic bond, which is detected in comet assay as single strand DNA breaks [18]. In fact, TMZ generates large amounts of FPG-sensitive sites on nuclear DNA. Furthermore, the combination of BSO with TMZ greatly potentiated TMZ-oxidized DNA lesions (Figure 2D). These results indicate that GSH acts as a protective cellular mechanism against TMZ, mitigating ROS induction, and also reducing, in turn, oxidized DNA damage originating from TMZ.

### NRF2 silencing potentiates TMZ cell death induction *in vitro*

**Figure 3:**
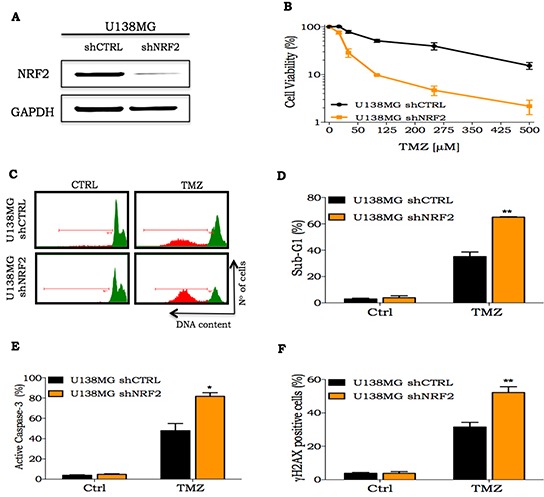
Cellular response of NRF2 silenced cells to TMZ treatment **A.** NRF2 detection by western blot in U138MG cells transduced with shCTRL or shNRF2 lentivirus; **B.** A dose-response curve of U138MG shCTRL or U138MG shNRF2 cell lines treated with increasing concentrations of TMZ (10 to 500 μM) and analyzed 72 h after drug treatment measured by XTT assay; **C-D.** Representative histogram and quantification of sub-G1 population of glioma cells treated with TMZ (100 μM) for 72 h, respectively; **E-F.** Flow cytometry analysis of percentage of active caspase-3 or γH2AX positive staining in cells NRF2 silenced or transduced with shCTRL upon treatment with TMZ (100 μM) for 72 h, respectively. Values are mean ± *SEM* of three independent experiments, *P< 0.05, **P< 0.01, ***P< 0.001.

To gain further insights concerning the role of NRF2 as a mediator of TMZ resistance, we established NRF2-silenced cell lines using an shRNA lentiviral system. As shown in Figure 3A there was a substantial decrease in NRF2 protein level in the U138MG shNRF2 cell line, when compared to U138MG shCTRL cells. A similar significant decrease was observed in the mRNA expression of NRF2, GCML and GSTπ in NRF2 depleted cells (Supplementary Figure S1). Notably, U138MG shNRF2 showed a greater sensitivity to TMZ treatment, as shown by the XTT cell viability assay (Figure 3B). Besides that, U138MG shNRF2 cells displayed a higher TMZ apoptosis induction than the shCTRL cell line. This is indicated by the increased sub-G1 population (Figure 3C-3D) and caspase-3 activation (Figure 3E). We also observed an increase in the amount of DNA damage induced by TMZ in shNRF2 cells in comparison to shCTRL cells, as seen by γH2AX positive cells (Figure 3F). Similar results were obtained with U87MG shNRF2 cells (data not shown). Due to notably difficulty of U138MG in forming tumor in *nude* mice, we performed *in vivo* procedures using U87MG cells.

### NRF2 silencing potentiate TMZ cell death induction *in vivo*

**Figure 4:**
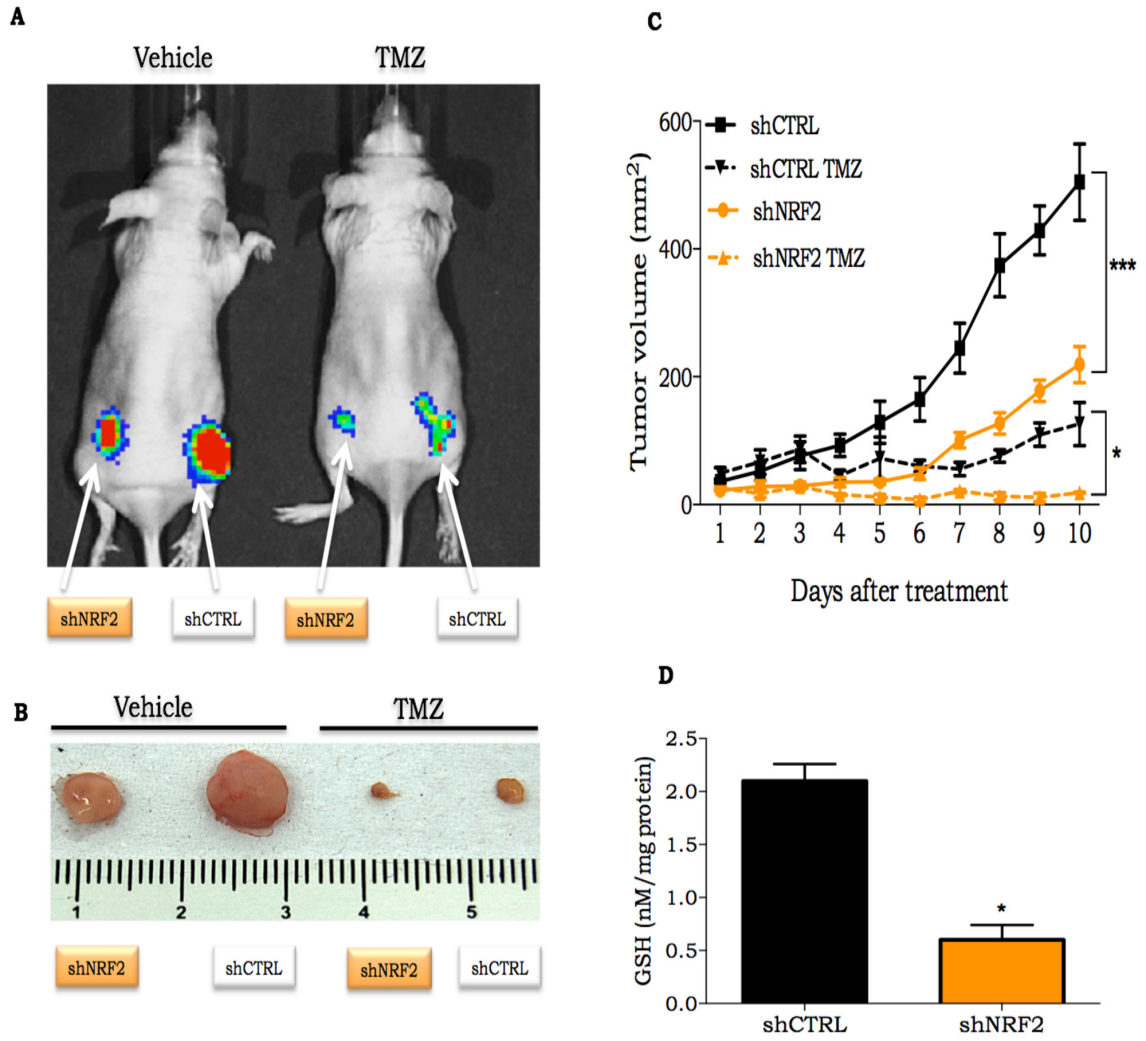
In vivo response of NRF2 silenced cells to TMZ treatment **A.** Representative bioluminescent image of shCTRL or shNRF2 expressing luciferase cells on day 10 after beginning treatment with TMZ (30 mg/kg); **B.** *Ex vivo* shCTRL or shNRF2 tumor at day 10 after initial TMZ treatment; **C.** Time-course of shCTRL or shNRF2 tumor volume progression, as determined by caliper measurement; **D.** Quantification of GSH concentration on shCRTL or shNRF2 tumors. Values are mean ± *SEM*; 5 animals were used per group.

The effects of NRF2 silencing were also experimentally tested *in vivo*. Female *nude* mice bearing U87MG shNRF2 and U87MG shCTRL cells on each side of the animal's flanks were submitted to vehicle (0.5% DMSO in PBS) or TMZ (30 mg/kg) treatment. A significant slower progression on shNRF2 tumors was observed, when compared to shCTRL tumor (Figure 4A-4C), even in the absence of any treatment. In addition, upon TMZ treatment, there was a greater inhibition of tumor growth on shNRF2 tumors when compared to shCTRL (Figure 4A-4C). Also, GSH and thiol levels measured on tumors were 4-fold lower in the shNRF2 cell line in comparison to control cells (Figure 4D and Supplementary Figure S2), indicating an inhibitory effect on GSH production in NRF2-depleted cells *in vivo*.

### GSH synthesis and GST inhibition potentiate TMZ cytotoxicity in glioma and melanoma cells

**Figure 5:**
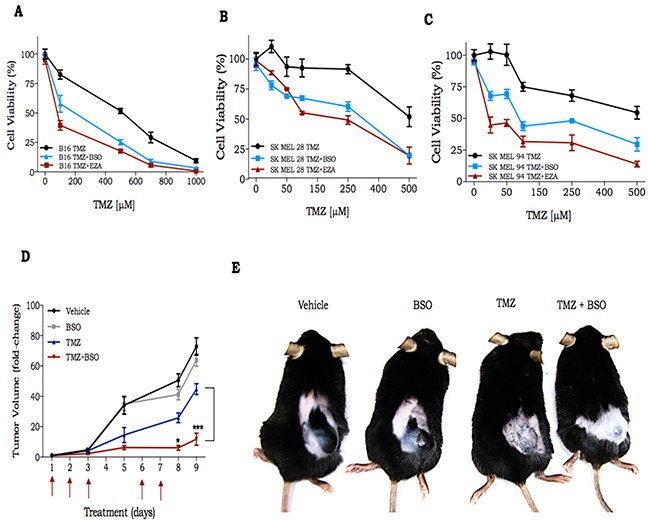
In vitro and in vivo response of melanoma cells to treatment with TMZ in combination with GSH modulators **A-C.** Dose response curve of murine and 2 human melanoma cell lines, respectively, to treatment with TMZ alone or in combination with BSO or EZA. Importantly, cell viability, for any of the cell lines, was not affected by BSO (blue line) or EZA (red line) in the absence of TMZ. Cell viability was measured 72 h after drug treatment by XTT assay; **D.** Time-course of B16Luc tumor volume progression, as determined by caliper measurement; **E.** Representative image of C57Bl/6 mice bearing B16Luc tumor at day 9 after treatment with TMZ (30 mg/kg) and/or BSO (450 mg/kg). Values are mean ± SEM ; 5 animals were used per group.

Patients with metastatic melanoma resistant to treatment with BRAF inhibitor or immunotherapy, are treated with DTIC, a TMZ analog [19]. Thus, we hypothesized that GSH could be involved in TMZ resistance in melanoma as well. Thereby, GSH modulation could enhance TMZ cytotoxicity in melanoma cells as we observed for glioma cells. U138MG glioma cell line and two human melanoma cell lines (SK MEL28, SK MEL94) and a murine melanoma cell line (B16) were treated with TMZ in combination to BSO or ezatiostat (EZA), a GST inhibitor. The concentrations of these substances (BSO or EZA) were such that no cytotoxicity was observed for any of the cell lines, in the absence of TMZ. Cell viability was evaluated three days after treatment and we observed that, in all cell lines, BSO as well as EZA were able to substantially potentiate TMZ cell killing effect (Figure 5A-5C and Supplementary Figure S3).

To investigate if the synergistic effect of TMZ combined with BSO also occurs *in vivo*, we inoculated B16 into C57Bl/6 animals divided into 4 experimental groups: 1) vehicle (0.5% DMSO in PBS); 2) BSO (450 mg/kg); 3) TMZ (30 mg/kg); 4) TMZ + BSO. B16 is well known by its high proliferative capacity and drug resistance [20]. We observed a measurable tumor 6 days after cell inoculation and at day 15 the animals were euthanized due to the high tumor burden (2,000 mm^3^). In fact, there was a 70-fold increase in tumor size compared to its initial volume in vehicle and BSO groups after 9 days (Figure 5D-5E). In the TMZ treated group, there was an approximate 40-fold increase in tumor volume, and we observed a remarkable reduction of tumor progression in the TMZ+BSO group (about 10-fold increase over the entire treatment period). Together these results indicate that GSH plays a central role in the promotion of TMZ resistance, not only in glioma, but also in melanoma cells.

## DISCUSSION

Over the last decades several cancer patients, such as those with testicular and breast tumors, truly beneficiated from antitumor drug development [21]. However, that is not the case for patients with glioma or metastatic melanoma, which face a dreadful prognosis even with all possible clinical therapeutic protocols available [22, 25].

Recently, the alkylating agent TMZ has received special attention, in particular for being one of few orally administered antitumor drugs, and for its ability to easily cross the blood brain barrier, a indispensable feature to treat brain tumors [24]. In fact, after being approved by the FDA in 2005, TMZ readily became the first line therapy to treat glioma [23]. However, glioma resistance towards TMZ cell death induction severely limited drug efficacy, and as consequence, this type of cancer remains incurable.

Several mechanisms are known to be involved on TMZ resistance. Most of them are related to DNA repair processes, which is not surprising given the fact that TMZ induces DNA damage [6]. In fact, many reports demonstrate a clear correlation between DNA repair mechanisms and TMZ resistance both *in vitro* and *in vivo* [26,27,28,29,30]. Despite a promising report on the use of a combination of TMZ and MGMT inhibitor O^6^-benzylguanine (O^6^-BG) [29], the outcomes of several clinical trials were not that encouraging. For instance, phase-II clinical trials using O^6^-BG showed no improvement on TMZ efficacy for patients with adult recurrent glioma [32] or pediatric high-grade glioma [31]. Thus, we reasoned that other important mechanisms could be involved in TMZ resistance.

Working with the U87MG cell line, Zhang et al. [8] observed a 2-fold increase in ROS production upon incubation with 250 μM TMZ for 2 h. Recently, it was demonstrated that TMZ, besides genomic DNA, also reacts with mitochondrial DNA [7] and Cai et al. showed that by targeting MGMT to the mitochondria, the cells are protected against TMZ [32]. Thus, mitochondrial malfunctioning could be the source of ROS production by TMZ. In the present study, we found an increase on mitochondrial ROS production after TMZ treatment. More importantly, we showed that NAC was able to completely inhibit ROS availability, and BSO significantly increased ROS levels in combination with TMZ. Furthermore, we observed a pronounced increase in oxidized DNA lesion after treatment with BSO plus TMZ, when compared to DNA damage levels generated by the drugs alone. Those results suggest a central role of GSH in mitigating ROS induced after treatment with TMZ.

In a previous work, we showed that the TMZ resistant cell line U138MG presented higher intracellular GSH levels than the more sensitive U87MG cells and drug resistance was abolished when TMZ was combined with BSO, both *in vitro* and *in vivo* [17]. In the present work, we described a significant difference between the two cell lines concerning NRF2 expression. In fact, U138MG cells presented significant higher levels of NRF2 as well as genes related to GSH synthesis and utilization than U87MG cells. These results are in agreement with our previous findings [17], and offer a reasonable explanation for the higher levels of GSH found in the resistant cell line.

Recently, it was demonstrated that NRF2 silencing in glioma cell lines inhibited cell proliferation [33], decreased cellular migration [34], and induced autophagic process [35]. Here we went further and showed that NRF2 silencing results in greater sensitivity and higher apoptosis induction upon TMZ treatment. Using an *in vivo* model, we also observed a pronounced decrease in tumor progression in NRF2 shRNA tumors on untreated animals, indicating that NRF2 plays a fundamental role on tumor growth rate. In addition, we found a significant decrease in GSH and thiol levels on NRF2 silenced tumors. More importantly, we observed an additive effect between TMZ treatment and NRF2-depletion. These results strongly indicate that GSH levels play a crucial role on TMZ resistance and also that GSH availability is tightly regulated by NRF2 in glioma cells.

In general, tumor cells present higher levels of GSH than normal ones and, as a consequence, they seem to be more dependent on GSH [36,37]. Thus, therapeutic strategies that modulate GSH levels are tempting therapeutic alternatives. BSO is an irreversible selective inhibitor of glutamate cysteine ligase (GCL), and its administration is able to deplete the GSH level by up to 90% in many cell lines both *in vitro* as well as *in vivo* [38]. Another interesting strategy is to disrupt GSH utilization by inhibiting GST enzymes, which could be done by incubation with EZA [39]. We found that the combination of BSO or EZA with TMZ greatly potentiated cell death induction of glioma cell lines *in vitro*.

Previous to TMZ, its analogue, DTIC was the most important methylating agent used in the clinic. DTIC was approved nearly 30 years ago and remains the reference drug to treat metastatic melanoma, even though complete response is achieved in less than 10% of patients [40]. DTIC needs to be metabolized in the liver in order to be activated and is unable to cross the blood brain barrier. Thus, DTIC is useless against brain metastasis, which is observed in about 60% of metastatic melanoma patients [4]. A phase III clinical trial comparing DTIC and TMZ showed that both drugs have equivalent impact on overall survival, but there was improvement on life quality in patients treated with TMZ [19]. Thus, TMZ is a valid therapeutic alternative to treat metastatic melanoma [41].

Based on that, we were interested to find out if TMZ resistance due to GSH availability was glioma specific or alternatively could be seen in other tumors, such as melanoma. In fact, combination of TMZ with either BSO or EZA had a profound impact on viability of melanoma cells in comparison to the cells treated with TMZ alone. The increased sensitivity to TMZ induced by EZA indicates that GSH may be acting as a detoxification agent towards TMZ. Furthermore, as previously mentioned, BSO in combination with TMZ was able to increase ROS production, which suggests that GSH is also protecting the tumor cells from TMZ as an antioxidative agent. Nevertheless, depletion of GSH using BSO is sufficient to inhibit both protective mechanisms (antioxidative and detoxification process), once it is limiting GSH availability. Remarkably, BSO plus TMZ substantially inhibited melanoma tumor progression *in vivo*.

**Figure 6:**
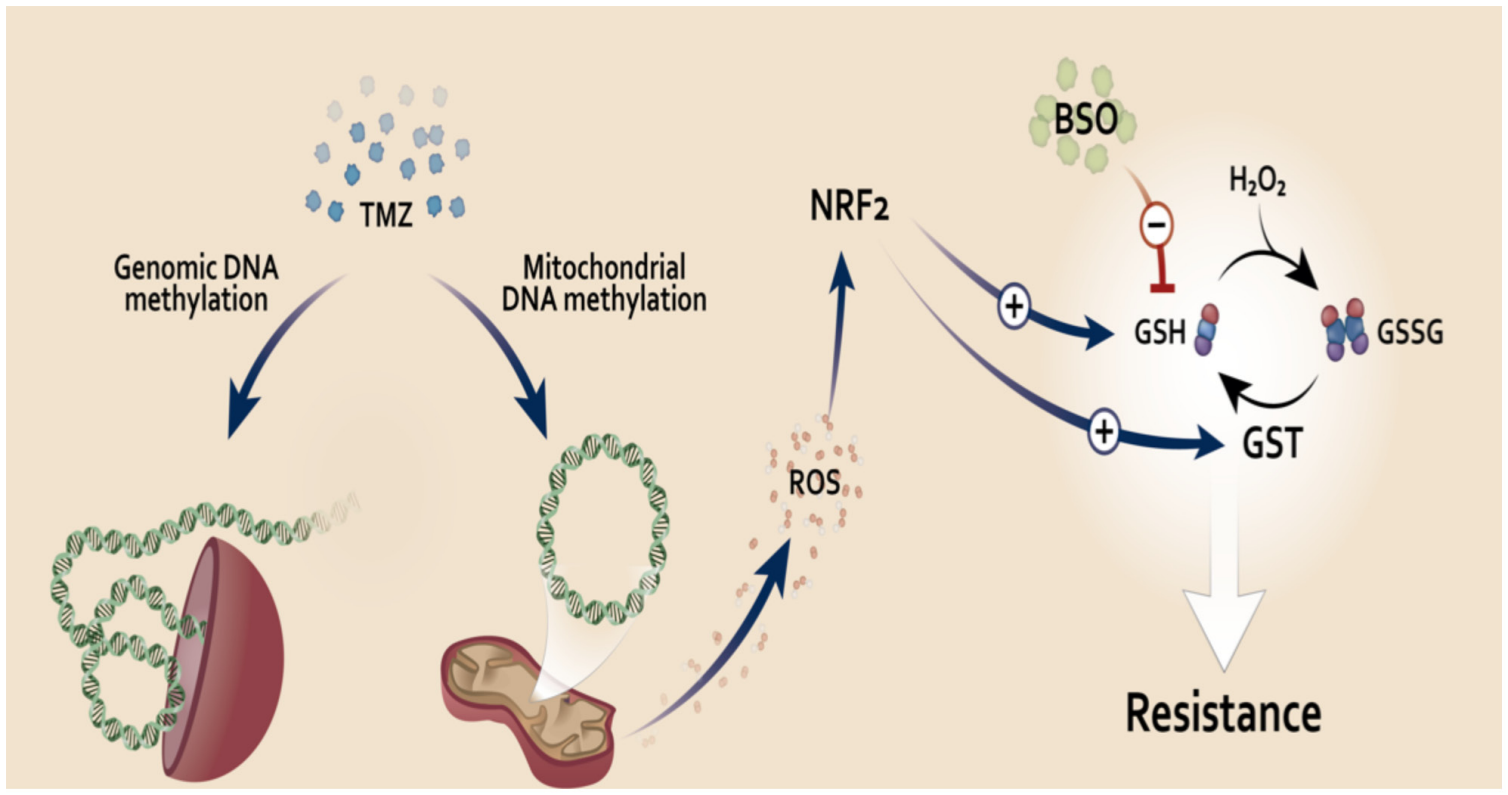
Proposed model of NRF2 role on TMZ resistance TMZ induces genomic and mitochondrial DNA methylation damage. Mitochondrial DNA damage could lead to malfunction of this organelle, increasing its ROS production, which in turn activates NRF2. This transcription factor induces expression of genes related to GSH synthesis and utilization. GSH could act as an antioxidant (neutralizing ROS induced upon TMZ treatment) or detoxification agent (by GSH binding to TMZ through GST activity, eliminating TMZ inside the cells). Increased NRF2 activity, leading to higher GSH levels, would be a key resistance mechanism to TMZ. Thus, we propose the use of the GSH inhibitor, BSO, in combination with TMZ to circumvent resistance to this drug in glioma and melanoma tumor.

In Figure 6 we summarize our findings, proposing a model to explain TMZ resistance mediated by NRF2. TMZ is an alkylating drug that inserts a methyl group on DNA bases. In this work, we showed that upon TMZ treatment there was an increase on ROS production due, at least in part, to mitochondria damage, which seems to be the trigger for the induction of NRF2 and consequently increase GCLM and GSTπ expression upon TMZ treatment. As a consequence, there is a significant increase of GSH availability and utilization, respectively, which, in turn, mediate TMZ resistance. Notably, we demonstrated that GSH depletion, using BSO, is responsible to circumvent TMZ drug resistance in glioma and melanoma cell lines.

Together our results indicate determinant roles of NRF2 in conferring TMZ tumor resistance mainly by induction of GSH synthesis and utilization. Thus, we propose that NRF2 is an important molecular marker to TMZ resistance and the use of BSO in combination with TMZ as an alternative therapeutic approach for fighting glioma and melanoma.

## MATERIALS AND METHODS

### Cell lines and culture conditions

Certified human glioma cell lines U87MG and U138MG; human melanoma cells lines SKMEL 28 and SKMEL 94 as well as murine melanoma B16 were kindly provided by Prof. Bernd Kaina, Germany. Human glioma and melanoma cells were routinely grown in DMEM (Invitrogen, Life Technologies, Carlsbad, CA, USA), and murine melanoma was grown in RPMI (Invitrogen). All culture media were supplemented with 10% FCS (fetal calf serum; Cultilab, Campinas, SP, Brazil) and 1% antibiotic-antimycotic at 37°C in a humidified, 5% CO_2_ atmosphere.

### Cell survival measurement

In a 12 multi-well plate, 2×10^4^ cells were plated and pre-treated for 16 h with 100 μM BSO (Sigma-Aldrich, St. Louis, MO, USA) or 1 mM NAC (Sigma-Aldrich), followed by incubation with increasing doses of TMZ for 72 h. After that, the cells were washed with phosphate-buffered saline (PBS) followed by incubation with XTT reagent kit as recommended by the manufacturer's instructions (Roche, Basel, Switzerland).

### Flow cytometry for sub-G1, active caspase-3 and γH_2_AX analysis

The apoptotic response after genotoxic drug treatment was measured using flow cytometry for sub-G1 determination. Supernatant and attached cells were collected, washed once with PBS and fixed in 70% ethanol. Ethanol-fixed cells were stained with propidium iodide (PI) at room temperature for 1 h in PBS containing 20 μg/ml PI (Sigma–Aldrich), 200 μg/ml RNase A, and 0.1% Triton X-100. The percentage of sub-G1 cells was calculated using the CytoSoft software (Millipore, Billerica, MA, USA). For γH_2_AX and active caspase-3 immunostaining, cells were fixed with 1% formaldehyde and then with 70% ethanol. Afterwards, the cells were blocked, permeabilized, incubated with either primary mouse monoclonal antibody to γH2AX (Ser-139) (Upstate Biotechnology, Lake Placid, NY, USA) and diluted 1:500, or mouse anti-active caspase 3 (BD, Pharmigen, San Diego, CA, USA) diluted 1:50 for 2 h at room temperature. This was followed by incubation with anti-mouse FITC secondary antibody (Sigma-Aldrich) that was diluted 1:200 for 1 h at room temperature. The percentage of γH_2_AX positive cells was again calculated using the CytoSoft software (Millipore).

### Analysis of reactive oxygen species (ROS) generation

ROS levels were analyzed using a DCFDA (2′,7′-dichlorofluorescein diacetate, Invitrogen) probe and Mitosox (Life Technologies), according to the manufaturer's protocol. Briefly, cells were detached with trypsin and incubated for 30 min with 10 μM of DCFDA or for 15 min with 5 μM of Mitosox in DMEM without phenol red containing 0.2% FBS. The mean green or red fluorescence intensity was measured by flow cytometry.

### Glutathione quantification

Intracellular GSH levels were quantified using the GSH-Glo Glutathione Assay (Promega), following the manufacturer's instructions. Briefly, 2×10^4^ cells were seeded in opaque, 96-well plates and allowed to grow for 24 h under cell culture conditions. Cells were washed with PBS and incubated for 30 min at room temperature in a solution containing luciferin NT substrate and glutathione-S-transferase. Then, 100 μL of luciferase enzyme was added, and incubated for 15 min at room temperature. Luminescence was measured using a Glomax-Multi+ Luminometer (Promega). Serial dilution of a GSH standard solution was used to generate a standard curve, and GSH concentration was normalized to the protein concentration of each well.

### Total reduced thiol levels

Total reduced thiols were determined in a spectrophotometer (Hitachi U-3300, Tokyo, Japan) using 5,5′-Dithiobis(2-nitrobenzoic acid) (DTNB). Thiol residues react with DTNB, cleaving the disulfide bond to give 2-nitro-5-thiobenzoate (NTB-), which ionizes to the NTB2- di-anion in water at neutral and alkaline pH. The NTB2- was quantified in a spectrophotometer by measuring the absorbance at 412 nm, and was expressed as nmol of reduced DTNB/mg protein [42].

### Alkaline comet assay

The alkaline comet assay was performed as previously described [43]. For detection of oxidized purines the Escherichia coli Formamido Pyrimidine–DNA Glycosylase (FPG) enzyme was used. Briefly, after overnight lysis, the comet slides were washed in FPG glycosylase buffer (40 mM Hepes pH 7, 10 mM MgCl_2_, 1 mM DTT), and incubated for 30 min at 37°C with FPG (New England Biolabs, Ipswich, MA, USA) at 8 U/ml in 1× NEBuffer 1, supplemented with 100 μg/ml BSA (both supplied by the manufacturer). One half of each slide was incubated with the enzyme, while the other half was incubated with the same solution, except for glycosylase (negative control). Comets were stained with ethidium bromide, imaged with a fluorescence microscope (Olympus BX51, Olympus, Center Valley, PA, USA), and at least 50 comets per slide were scored for each condition.

### Western blot

Cells were lysed and cell protein extraction was quantified using Pierce BCA Protein Assay kit (Thermo Scientific, Rockford, IL, EUA). Proteins were separated on an SDS–polyacrylamide gel and blotted onto a nitrocellulose transfer membrane (GE Healthcare, Waukesha, WI, USA). Membranes were blocked for 1 h in 5% (w/v) milk powder in PBS, and incubated overnight at 4°C with primary antibody against anti-NRF2 (1:500) and anti-GAPDH (1:2000) (Santa Cruz Biotechnology, Santa Cruz, CA, USA). A chemiluminescent HRP substrate (Millipore) was used to develop the membranes, and the luminescence intensity was determined using an ImageQuant 300 (GE Healthcare).

### Real-time PCR

Total RNA was extracted using PureLink RNA Mini kit (Invitrogen), following the manufacturer's protocol. After DNase (Promega, Madison, WI, USA) treatment, cDNA was prepared using a High Capacity cDNA Reverse Transcription kit (Applied Biosystems, Life Technologies). Gene expression was determined by real-time quantitative PCR (Q-PCR). Briefly, 3 μL of diluted cDNA, 6 μL of SYBR green master mix, 0.5 μL of 10 mM forward and reverse primers and nuclease-free water were used in a combined total volume of 12 μL for each reaction. Q-PCR was carried out using the 7500 Real-Time PCR System (Applied Biosystems). The relative expression levels of the genes of interest were calculated using the relative standard curve method, based on the individual Q-PCR primer efficiencies, and the quantified values were normalized against the housekeeping gene encoding GAPDH.

### Establishment of glioma cells expressing luciferase and depleted for NRF2

pLV/Luc lentiviral vector was generated as previously described [17]. This plasmid was co-transfected with three auxiliary plasmids into HEK 293FT cells using the polyethyleneimine (PEI) method. The recombinant lentivirus was then used to transduce U87MG shCTRL or U87MG shNRF2, resulting in the stable-expressing luciferase glioma cell lines (Luc cells).

### *In vivo* procedures

Xenograft tumors were established in 10-12-week-old, female, athymic nude mice. U87MG shCTRL or U87MG shNRF2 Luc cells (3×10^6^) were inoculated subcutaneously in the animal's flank. Tumors were allowed to grow, and approximately 3 weeks after inoculation, treatment began. Tumor volume was calculated according to the following formula: volume = (width^2^ × length)/2. Animals were randomized into 2 treatment groups: (1) vehicle (0.5% DMSO diluted on PBS); (2) TMZ (30 mg/kg). TMZ was injected i.p. for 3 consecutive days, followed by a 2-day interval and subsequent 3 consecutive days of drug treatment.

B16 cells (10^5^) were injected into C57Bl/6 mice The animals were randomized into 4 treatment groups: (1) vehicle BSO was inoculated i.p. 5 h before TMZ treatment for three consecutive days. All animal procedures were approved by the Ethics Committee for Animal Care and Use of the Institute of Biomedical Sciences, University of Sao Paulo.

### Bioluminescence imaging

For *in vivo* luciferase assays, 150 mg/kg D-luciferin (Promega, Madison, WI, USA) was inoculated i.p. into nude mice to measure the tumor size. Bioluminescence images were obtained using the IVIS Spectrum system (Perkin-Elmer Life Sciences, Waltham, MA, USA) at the CEFAP-USP facility.

### Statistical analysis

Results represent the mean of three independent experiments, each performed in triplicate, with error bars showing the standard error of the mean (SEM). Statistical significance among data sets was accessed by applying one-way ANOVA followed by Bonfferoni post-testing (Prism 6 –GraphPad Software Inc., CA, USA) (*P<0.05, **P<0.01, ***P<0.001).

## SUPPLEMENTARY FIGURES



## References

[1] Stupp R, Tonn JC, Brada M, Pentheroudakis G (2010). High-grade malignant glioma: ESMO Clinical Practice Guidelines for diagnosis, treatment and follow-up. Ann. Oncol.

[2] Wen PY, Kesari S (2008). Malignant gliomas in adults. N. Engl. J. Med.

[3] van den Bent MJ, Hegi ME, Stupp R (2006). Recent developments in the use of chemotherapy in brain tumours. Eur. J. Cancer.

[4] Fonkem E, Uhlmann E, Floyd EJ, Mahadevan SR, Kasper A, Eton E, Wong ET (2012). Melanoma brain metastasis: overview of current management and emerging targeted therapies. Expert Rev. Neurother.

[5] Schadendorf D, Fisher D, Garbe DE, Gershenwald C, Grob JE, Halpern J, Herlyn A, Marchetti M, McArthur MA, Ribas G, Roesch A, Hauschild A (2015). Melanoma. Nat. Rev. Dis. Prim.

[6] Johannessen TC, Bjerkvig R (2012). Molecular mechanisms of temozolomide resistance in glioblastoma multiforme. Expert Rev. Anticancer Ther.

[7] Wang H, Cai S, Ernstberger A, Bailey BJ, Wang MZ, Cai W, Goebel WS, Czader MB, Crean C, Suvannasankha A, Shokolenkoc I, Wilson GL, Baluyut AR (2013). Temozolomide-Mediated DNA Methylation in Human Myeloid Precursor Cells: Differential Involvement of Intrinsic and Extrinsic Apoptotic Pathways. Clin. Cancer Res.

[8] Zhang W, Wang Z, Shu F, Jin Y, Liu H, Wang Q, Yang Y (2010). Activation of AMP-activated Protein Kinase by Temozolomide Contributes to Apoptosis in Glioblastoma Cells via p53 Activation and mTORC1 Inhibition. J. Biol. Chem.

[9] Ma Q (2013). Role of Nrf2 in Oxidative Stress and Toxicity. Annu. Rev. Pharmacol. Toxicol.

[10] Jaramillo MC, Zhang DD (2013). The emerging role of the Nrf2–Keap1 signaling pathway in cancer. Genes Dev.

[11] Sporn MB, Liby KT (2012). NRF2 and cancer: the good, the bad and the importance of context. Nat. Rev. Cancer.

[12] Zhang H, Forman HJ (2012). Glutathione synthesis and its role in redox signaling. Semin. Cell Dev. Biol.

[13] Harvey CJ, Thimmulappa RK, Singh A, Blake DJ, Ling G, Wakabayashi N, Fujii J, Myers A, Biswal S (2009). Nrf2-regulated glutathione recycling independent of biosynthesis is critical for cell survival during oxidative stress. Free Radic. Biol. Med.

[14] Chen HHW, Kuo MT (2010). Role of Glutathione in the Regulation of Cisplatin Resistance in Cancer Chemotherapy. Met. Based. Drugs.

[15] Townsend DM, Tew KD (2003). The role of glutathione-S-transferase in anti-cancer drug resistance. Oncogene.

[16] Byun SS, Kim SW, Choi H, Lee C, Lee E (2005). Augmentation of cisplatin sensitivity in cisplatin-resistant human bladder cancer cells by modulating glutathione concentrations and glutathione-related enzyme activities. BJU Int.

[17] Rocha CRR (2015). Garcia CCM, Vieira DB, Quinet A, de Andrade-Lima LC, Munford V, Belizario JE, Menck CFM. Glutathione depletion sensitizes cisplatin- and temozolomide-resistant glioma cells in vitro and in vivo. Cell Death Dis.

[18] Prakash A, Doublié S, Wallace SS (2012). The Fpg/Nei Family of DNA Glycosylases: Substrates, Structures, and Search for Damage. Mech. DNA Repair.

[19] Middleton MR, Grob JJ, Aaronson N, Fierlbeck G, Tilgen W, Seiter S, Gore M, Aamdal S, Cebon J, Coates A, Dreno B, Henz M, Schadendorf D (2000). Randomized Phase III Study of Temozolomide Versus Dacarbazine in the Treatment of Patients With Advanced Metastatic Malignant Melanoma. J. Clin. Oncol.

[20] Ortega A, Ferrer P, Carretero J, Obrador E, Asensi M, Pellicer JA, Estrela JM (2003). Down-regulation of Glutathione and Bcl-2 Synthesis in Mouse B16 Melanoma Cells Avoids Their Survival during Interaction with the Vascular Endothelium. J. Biol. Chem.

[21] Siegel RL, Miller KD, Jemal A (2015). Cancer statistics, 2015. Cancer J. Clin.

[22] Newlands ES, Stevens MFG, Wedge SR, Wheelhouse RT, Brock C (1997). Temozolomide: a review of its discovery, chemical properties, pre-clinical development and clinical trials. Cancer Treat. Rev.

[23] Stupp R, Mason WP, van den Bent MJ, Weller M, Fisher B, Taphoorn MJB, Belanger K, Brandes AA, Marosi C, Bogdahn U, Curschmann J, Janzer RC, Ludwin SK (2005). Radiotherapy plus Concomitant and Adjuvant Temozolomide for Glioblastoma. N. Engl. J. Med.

[24] McFaline-Figueroa JL, Braun CJ, Stanciu M, Nagel ZD, Mazzucato P, Sangaraju D, Cerniauskas E, Barford K, Vargas A, Chen Y, Tretyakova N, Lees JA, Hemann MT (2015). Minor Changes in Expression of the Mismatch Repair Protein MSH2 Exert a Major Impact on Glioblastoma Response to Temozolomide. Cancer Res.

[25] Hegi ME, Diserens AC, Gorlia T, Hamou MF, de Tribolet N, Weller M, Kros JM, Hainfellner JA, Mason W, Mariani L, Bromberg JE, Hau P, Mirimanoff RO (2005). MGMT gene silencing and benefit from temozolomide in glioblastoma. N Engl J Med.

[26] Agnihotri S, Gajadhar AS, Ternamian C, Gorlia T, Diefes KL, Mischel PS, Kelly J, McGown G, Thorncroft M, Carlson BL, Sarkaria JN, Margison GP, Aldape K (2012). Alkylpurine–DNA–N-glycosylase confers resistance to temozolomide in xenograft models of glioblastoma multiforme and is associated with poor survival in patients. J. Clin. Invest.

[27] Kaina B, Christmann M, Naumann S, Roos WP (2007). MGMT: Key node in the battle against genotoxicity, carcinogenicity and apoptosis induced by alkylating agents. DNA Repair.

[28] Roos WP, Batista LFZ, Naumann SC, Wick W, Weller M, Menck CFM, Kaina B (2007). Apoptosis in malignant glioma cells triggered by the temozolomide-induced DNA lesion O6-methylguanine. Oncogene.

[29] Koch D, Hundsberger T, Boor S, Kaina B (2007). Local intracerebral administration of O6-benzylguanine combined with systemic chemotherapy with temozolomide of a patient suffering from a recurrent glioblastoma. J. Neurooncol.

[30] Quinn JA, Jiang SX, Reardon DA, Desjardins A, Vredenburgh JJ, Rich JN, Gururangan S, Friedman AH, Bigner DD, Sampson JH, McLendon RE, Herndon JE, Walker A (2009). Phase II Trial of Temozolomide Plus O6-Benzylguanine in Adults With Recurrent, Temozolomide-Resistant Malignant Glioma. J. Clin. Oncol.

[31] Warren KE, Gururangan S, Geyer JR, McLendon RE, Poussaint TY, Wallace D, Balis FM, Berg SL, Packer RJ, Goldman S, Minturn JE, Pollack IF, Boyett JM (2011). A phase II study of O6-benzylguanine and temozolomide in pediatric patients with recurrent or progressive high-grade gliomas and brainstem gliomas: a Pediatric Brain Tumor Consortium study. J. Neurooncol.

[32] Cai S, Xu Y, Cooper RJ, Ferkowicz MJ, Hartwell JR, Pollok KE, Kelley MR (2005). Mitochondrial Targeting of Human O6-Methylguanine DNA Methyltransferase Protects against Cell Killing by Chemotherapeutic Alkylating Agents. Cancer Res.

[33] Ji X, Chen S, Zhu L, Pan H, Zhou Li, Wang W (2013). Knockdown of NF-E2-related factor 2 inhibits the proliferation and growth of U251MG human glioma cells in a mouse xenograft model. Oncol. Rep.

[34] Pan H, Wang H, Zhu Lin, Mao L, Qiao L, Su X (2013). The Role of Nrf2 in Migration and Invasion of Human Glioma Cell U251. World Neurosurg.

[35] Zhou Y, Wang H, Zhu L, Cong Z, Li N, Ji X, Li W (2013). Knockdown of Nrf2 enhances autophagy induced by temozolomide in U251 human glioma cell line. Oncol. Rep.

[36] Gorrini C, Harris IS, Mak TW (2013). Modulation of oxidative stress as an anticancer strategy. Nat Rev Drug Discov.

[37] Hecht F, Pessoa CF, Gentile LB, Rosenthal D, Carvalho DP, Fortunato RS (2016). The role of oxidative stress on breast cancer development and therapy. Tumor Biol.

[38] Griffith OW (1982). Mechanism of action, metabolism, and toxicity of buthionine sulfoximine and its higher homologs, potent inhibitors of glutathione synthesis. J. Biol. Chem.

[39] Raza A, Galili N, Smith S, Godwin J, Lancet J, Melchert M, Jones M, Keck JG, Meng L, Brown GL, List A (2009). Phase 1 multicenter dose-escalation study of ezatiostat hydrochloride (TLK199 tablets), a novel glutathione analog prodrug, in patients with myelodysplastic syndrome. Blood.

[40] Luke JJ, Schwartz GK (2013). Chemotherapy in the management of advanced cutaneous malignant melanoma. Clin. Dermatol.

[41] Quirbt I, Verma S, Petrella T, Bak K, Charette M (2007). Temozolomide for the treatment of metastatic melanoma. Curr. Oncol.

[42] Ellman GL (1959). Tissue sulfhydryl groups. Arch. Biochem. Biophys.

[43] Vessoni AT, Quinet A, de Andrade-Lima LC, Garcia DJ, Machado CC, Rocha CRR, Vieira DB, Menck CFM (2016). Chloroquine-induced glioma cells death is associated with mitochondrial membrane potential loss, but not oxidative stress. Free Radic. Biol. Med.

