# Supplementary material for: NRF2 and glutathione are key resistance mediators to temozolomide in glioma and melanoma cells
Source: Oncotarget. 2016 Jun 17;7(30):48081–92. doi: 10.18632/oncotarget.10129 (PMC5217002; doi:10.18632/oncotarget.10129)
Supplement: Supplementary file 1 [file oncotarget-07-48081-s001.pdf]

NRF2 and glutathione are key resistance mediators to temozolomide in glioma and melanoma cells

SUPPLEMENTARY FIGURES

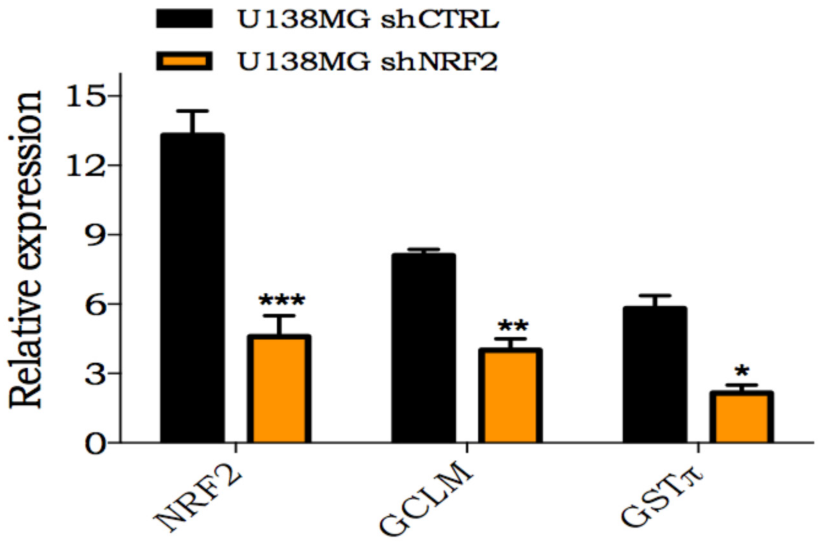

Supplementary Figure S1: Expression of NRF2 and its target genes in shCTRL or shNRF2 U138MG cell line. Quantification of NFR2, GCLM and GSTπ mRNA in U138MG shCTRL and U138MG shNRF2 at basal level. Values are mean ± *SEM* of three independent experiments, *P< 0.05, **P< 0.01, ***P< 0.001.

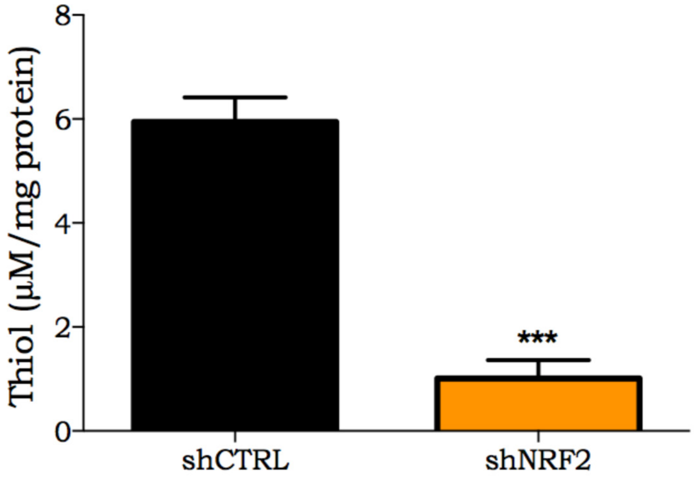

Supplementary Figure S2: Thiol levels of NRF2 depleted tumor cells. Quantification of thiol concentration on U87MG shCRTL or shNRF2 tumors. Values are mean ± *SEM;* 5 animals were used per group, *P< 0.05, **P< 0.01, ***P< 0.001.

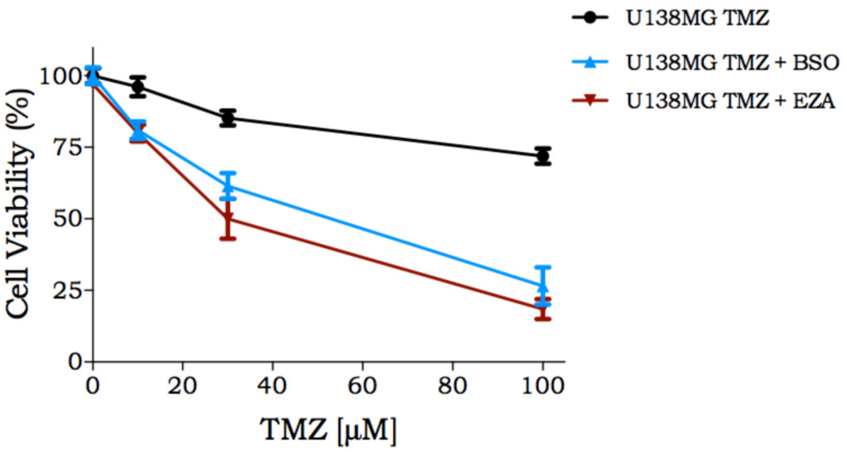

Supplementary Figure S3: *In vitro* response of glioma cells to treatment with TMZ in combination with GSH modulators. Dose response curve of U138MG glioma cells line to treatment with TMZ alone or in combination with BSO or EZA. Importantly, cell viability of these cells was not affected by BSO (blue line) or EZA (red line) in the absence of TMZ (0 µM TMZ). Cell viability was measured 72 h after drug treatment by XTT assay.
